# Sex-related differences in adult patients with status epilepticus: a seven-year two-center observation

**DOI:** 10.1186/s13054-023-04592-6

**Published:** 2023-08-05

**Authors:** Sira M. Baumann, Pia De Stefano, Paulina S. C. Kliem, Pascale Grzonka, Caroline E. Gebhard, Oana E. Sarbu, Gian Marco De Marchis, Sabina Hunziker, Stephan Rüegg, Andreas Kleinschmidt, Jérôme Pugin, Hervé Quintard, Stephan Marsch, Margitta Seeck, Raoul Sutter

**Affiliations:** 1 Clinic for Intensive Care Medicine, Department of Acute Care, University Hospital Basel, Basel, Switzerland; 2 Neuro-Intensive Care Unit, Department of Intensive Care, University Hospital of Geneva, Geneva, Switzerland; 3 EEG & Epilepsy Unit, Department of Clinical Neurosciences, University Hospital of Geneva, Geneva, Switzerland; 4 Department of Neurology, University Hospital Basel, Basel, Switzerland; 5 Medical Faculty of the University of Basel, Basel, Switzerland; 6 Medical Communication and Psychosomatic Medicine, University Hospital Basel, Basel, Switzerland; 7 Department of Clinical Research, University of Basel, Basel, Switzerland; 8 Medical Faculty of the University of Geneva, Geneva, Switzerland

**Keywords:** Status epilepticus, Sex, Neurocritical care

## Abstract

**Background:**

Conflicting findings exist regarding the influence of sex on the development, treatment, course, and outcome of status epilepticus (SE). Our study aimed to investigate sex-related disparities in adult SE patients, focusing on treatment, disease course, and outcome at two Swiss academic medical centers.

**Methods:**

In this retrospective study, patients treated for SE at two Swiss academic care centers from Basel and Geneva from 2015 to 2021 were included. Primary outcomes were return to premorbid neurologic function, death during hospital stay and at 30 days. Secondary outcomes included characteristics of treatment and disease course. Associations with primary and secondary outcomes were assessed using multivariable logistic regression. Analysis using propensity score matching was performed to account for the imbalances regarding age between men and women.

**Results:**

Among 762 SE patients, 45.9% were women. No sex-related differences were found between men and women, except for older age and lower frequency of intracranial hemorrhages in women. Compared to men, women had a higher median age (70 vs. 66, *p* = 0.003), had focal nonconvulsive SE without coma more (34.9% vs. 25.5%; *p* = 0.005) and SE with motor symptoms less often (52.3% vs. 63.6%, *p* = 0.002). With longer SE duration (1 day vs. 0.5 days, *p* = 0.011) and a similar proportion of refractory SE compared to men (36.9% vs. 36.4%, *p* = 0.898), women were anesthetized and mechanically ventilated less often (30.6% vs. 42%, *p* = 0.001). Age was associated with all primary outcomes in the unmatched multivariable analyses, but not female sex. In contrast, propensity score-matched multivariable analyses revealed decreased odds for return to premorbid neurologic function for women independent of potential confounders. At hospital discharge, women were sent home less (29.7% vs. 43.7%, *p* < 0.001) and to nursing homes more often (17.1% vs. 10.0%, *p* = 0.004).

**Conclusions:**

This study identified sex-related disparities in the clinical features, treatment modalities, and outcome of adult patients with SE with women being at a disadvantage, implying that sex-based factors must be considered when formulating strategies for managing SE and forecasting outcomes.

**Supplementary Information:**

The online version contains supplementary material available at 10.1186/s13054-023-04592-6.

## Background

Status epilepticus (SE) is a critical and life-threatening neurological condition with ongoing epileptic seizures [1] that comes along with a high morbidity and mortality [2]. This has triggered several studies to explore the impact of various demographic, clinical, epileptological, and treatment characteristics on the course and outcome of critically ill patients with SE. Several factors have been identified that may influence the development, management, course, and outcome of SE, including age, etiology, underlying comorbidities, type of seizures, duration of SE, and treatment modalities. In contrast, studies regarding the role of sex on the emergence of SE, its treatment, course, and outcome are scarce. Although some studies have suggested that female sex may be a risk factor for the development of SE in patients with epilepsy [3], and that women may receive less aggressive care than men regardless of illness severity [4], other studies have reported conflicting findings. For example, early studies suggested that the incidence of SE was lower in women than men, and other studies found no significant sex-related differences regarding the incidence of SE [5–8]. Given these inconsistences and further conflicting data from population-based studies and systematic reviews regarding the influence of sex on course and outcome in patients suffering from SE, the precise role of sex in this regard remains unclear [7, 9–12]. Hence, further research is needed to elucidate the role of sex in the management of SE. In particular, there is a need for more comprehensive studies that examine sex-associated differences in SE patients, including their demographics, clinical characteristics, disease course, and outcome, as well as differences especially when it comes to the development and implementation of treatment strategies. This retrospective observational cohort study aimed to investigate sex-associated differences in adult patients with SE, including treatment, course of disease, complications, and outcomes. 

## Methods

The retrospective study was performed at the University Hospitals of Basel and Geneva, two Swiss tertiary academic medical care centers. The STROBE-guidelines were followed to enhance the quality of reporting [13]. In accordance with the 1964 Declaration of Helsinki and its subsequent revisions, the local ethics committees (EKNZ 2019–00693 for Basel and CCER 2019–00836 for Geneva) granted approval for the study. The requirement for obtaining patient consent was waived.

### Data collection

The clinical data of both medical care centers were collected following the registered STEP-UP study (ClinicalTrials.gov ID: NCT04204863) previously initiated at the University Hospital of Basel and collecting clinical and electrophysiologic data of adult (≥ 18 years of age) patients with SE. From January 1st, 2015 to December 31st, 2021, clinical, laboratory and epileptologic data of all consecutive patients were collected and data were extracted from digital medical records and managed with the password encrypted online browser-based, metadata-driven database organizer REDCAP (Research Electronic Data Capture) [14]. Patients with SE following hypoxic-ischemic encephalopathy (HIE) were excluded from the study as HIE-induced SE has been previously established as a distinct and independent clinical entity known to be associated with elevated mortality rates.

The following data were collected: age, sex, presumed etiology of SE (categorized as potential non-fatal and fatal etiologies as defined elsewhere [15]), and the Glasgow Coma Score (GCS) at SE onset. The types of SE were determined by evaluating the digital EEG reports and/or emergency medical service reports. SE was categorized into the following predefined types as recommended by the current guidelines of the International League Against Epilepsy (ILAE) [1]: focal nonconvulsive without coma (with or without altered consciousness and absences), with motor symptoms (myoclonic and convulsive), and nonconvulsive with coma. Illness severity was quantified by the Status Epilepticus Severity Score (STESS; range 0–6) [16, 17], the Charlson Comorbidity Index (range 0–37) [18], and the Simplified Acute Physiology Score II (SAPS II; range 0–163) [19]. SE duration was defined as the time-period between the diagnosis of SE and the clinical and/or electroencephalographic evidence of seizure termination as previously described [20]. For patients with refractory SE who were treated with anesthesia to achieve an EEG burst-suppression pattern, the duration of SE was determined as the period from seizure onset until the establishment of burst-suppression, if the patient showed no relapse into SE after weaning of anesthetics.

In both centers, patients with SE were monitored with continuous EEGs or intermittent spot EEGs. Continuous EEGs were performed daily for at least 12 h per day and spot EEGs for at least 30 min every 12 h. Thereby, the calculated SE duration represents an approximation with a maximum inaccuracy of 12 h.

The following treatment characteristics were assessed: admission via emergency medical services and time from alarm to hospital admission, duration of mechanical ventilation, the number of administered non-sedating antiseizure drugs, continuously administered anesthetics, and vasopressors administered during SE, as well as duration of in-hospital treatment and ICU stay in days. Finally, complications during SE were noted including infections, arterial hypotension requiring the use of continuously administered vasopressors, and organ failure. In addition, care withdrawal and destination at hospital discharge were extracted from the medical records. To ensure consistency across the datasets all clinical, laboratory, and epileptologic data from patients with non-refractory and refractory SE were collected using the same methodologies.

### Antiseizure treatment

During the study period, the antiseizure treatment protocol for SE involved a stepwise approach following the guidelines of the American Epilepsy Society and the Neurocritical Care Society and were guided by the same neurologists and neurointensivists [21, 22]. The first-line treatment consisted of an intravenous benzodiazepine bolus, which was repeated if seizures persisted. For SE not responding to intravenously administered benzodiazepine boluses, second-line treatment was started, which included levetiracetam, lacosamide, valproic acid, or phenytoin. For patients with SE refractory to first- and second-line antiseizure treatment, continuously administered anesthetics were started as the third-line treatment, including propofol and midazolam. In addition, non-sedating antiseizure drugs were added, such as topiramate, zonisamide, oxcarbazepine, pregabalin, sultiame, or perampanel. As part of routine practice, anesthetics were titrated upon the discretion of the treating physicians with the aim of achieving a persistent electrographic proof of either seizure cessation or a burst-suppression pattern for at least 24 h [23]. If SE reoccurred after weaning of third-line anesthetic treatment, barbiturates were started and titrated to induce a complete burst-suppression or an isoelectric EEG.

### Outcomes

Primary outcomes were return to premorbid neurologic function, death in-hospital and on day 30 after SE onset. Return to premorbid neurologic function was defined as full recovery of all patient’s neurological abilities or restoration to the neurologic functioning present prior to SE based on the physicians’ notes at hospital discharge (as routinely documented in SE patients).

Secondary outcomes included treatment characteristics and course of disease.

### Statistics

Patients were categorized according to sex. Chi-square and Fisher exact test, where appropriate, were used for univariable comparisons of proportions. Continuous variables were compared using the Mann–Whitney* U* test or the Kruskal–Wallis test. Discrete variables were expressed as counts and percentages, and continuous variables were expressed as medians and interquartile ranges (IQR). The level of significance for multiple univariable analyses was adjusted using the Bonferroni correction for multiple comparisons. Uni- and multivariable logistic regression models were performed to identify associations between sex and all primary outcomes (i.e., return to premorbid neurologic function at discharge, death during hospital stay and at 30 days after SE onset). To correct for potential confounding, all baseline characteristics differing between sex and potentially related to outcome, were included into the multivariable logistic regression models. In addition, well-established outcome determinants (based on the current literature [24]), such as SE severity as quantified by the STESS, were included into the multivariable models independent of their distribution between sex in our cohort. To cheque for linearity between our continuous variable “age” and our primary outcomes, we performed the Box-Tidwell test by adding log-transformed interaction terms between age and its corresponding natural log into the model. Insignificance of the *p* values of the interaction terms implies a linear relation to the logit of the outcome variables confirming that the assumption is satisfied. To further explore associations between sex and differences in SE treatment (defined as secondary outcomes), both uni- and multivariable logistic regression models were performed, the latter including all baseline characteristics differing between sex in univariable comparisons.

To account for the imbalances regarding age between men and women, an additional analysis was performed using propensity score matching. The probability of being female (i.e., propensity score) was calculated with a logistic regression model based on age. Female and male patients were matched on propensity score with use of nearest-neighbor matching. Patients without an eligible match were excluded from additional analyses to reduce the risk of bias from non-exchangeable subjects. The Mann–Whitney U test was used to check for propensity score and age-balances between females and males in the matched cohort. The same uni- and multivariable logistic regression models as performed for the unmatched cohort were repeated to calculate associations with the primary outcomes in the propensity matched cohort. Propensity scores and respective matching was performed using "PSMATCH2: Stata module to perform full Mahalanobis and propensity score matching, common support graphing, and covariate imbalance testing" (http://ideas.repec.org/c/boc/bocode/s432001.html.), version 4.0.12 30jan2016 by E. Leuven, B. Sianesi.

For multivariable logistic regression models, the Hosmer–Lemeshow chi-square goodness-of-fit tests were performed. These tests provide summary measures of calibration based upon a comparison of observed and estimated outcomes [25]. All multivariable models were adjusted for the potential influence of the participating centers. For all multivariable models, a two-sided p values ≤ 0.05 were considered significant.

Statistical analysis was performed with STATA®16.1 (Stata Corp., College Station, TX, USA).

## Results

### Univariable comparisons of baseline characteristics

**Fig. 1:**
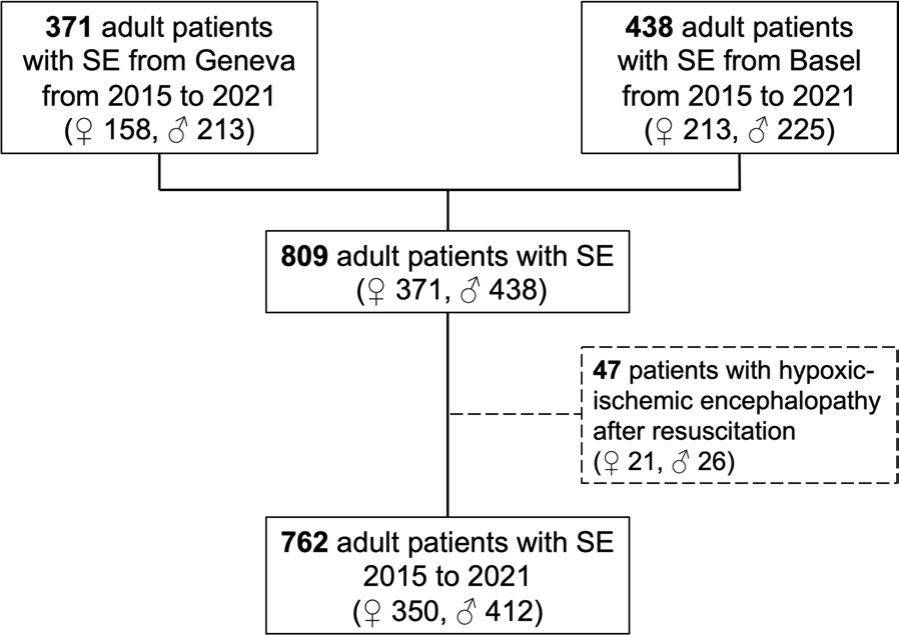
Flow chart. SE = status epilepticus; ♀ = female patients; ♂ = male patients

Among 762 SE patients (371 treated in Geneva, 438 treated in Basel), 45.9% were women (flow chart; Fig. 1). Univariable comparisons of demographics and clinical characteristics between men and women are presented in Table 1. Compared to men, women had a higher median age, a higher proportion of focal nonconvulsive SE (NCSE) without coma, and a lower proportion of SE with motor symptoms. EEG data were not available in 9.8% of patients, either because EEG was not performed due to rapid cessation of SE and full recovery (7.7%) or due to recording errors (2.1%). While most presumed underlying etiologies of SE were equally distributed, acute intracerebral hemorrhages was diagnosed as the presumed underlying etiologies of SE less often in women than in men.

**Table 1:** Univariable comparisons of baseline characteristics between men and women with status epilepticus (*n* = 762)

Baseline characteristics	Women (*n* = 350)		Men (*n* = 412)		
Demographics and clinical characteristics	*n*/median	%/IQR	*n*/median	%/IQR	p value
Age (years; median, IQR)	70	56–81	66	53–76	**0.003**
Out-of-hospital SE (i.e., admitted for SE; *n*, %)	292	83.4	355	86.2	0.293
Admitted via other hospital (*n*, %)	63	18	60	14.6	0.199
*SE etiology (n*, *%)*					
Presumed fatal etiology (not mutually exclusive)	92	26.3	107	26.0	0.921
Fast growing brain tumors	50	14.3	60	14.6	0.914
Acute intracranial hemorrhage	31	8.9	63	15.3	**0.007**
Infectious (meningo-)encephalitis	15	4.3	16	3.9	0.855
Acute ischemic stroke	11	3.1	10	2.4	0.658
Acute severe traumatic brain injury	7	2.0	23	5.6	0.014
Acute autoimmune encephalitis*	13	3.7	9	2.2	0.278
Presumed non-fatal etiology**	258	73.7	305	74.0	0.921
Known epilepsy	115	32.9	150	36.4	0.305
Unknown etiology	37	10.6	29	7.0	0.084
*Consciousness at SE onset*					
GCS at SE onset (median, IQR)	9	5–13	8	4–12	0.073
Coma at SE onset (*n*, %)	127	36.3	174	42.2	0.094
*SE type (n, %)*					
Focal NCSE without coma	122	34.9	105	25.5	**0.005**
With altered consciousness	93	26.6	75	18.2	**0.005**
Without altered consciousness	29	8.3	30	7.3	**0.005**
SE with motor symptoms (convulsive or myoclonic)	183	52.3	262	63.6	**0.002**
Convulsive SE	121	34.6	188	45.6	**0.002**
Myoclonic SE	62	17.7	74	18.0	0.929
NCSE with coma	45	12.9	45	10.9	0.410
NCSE with coma (non-subtle)	32	9.1	26	6.3	0.117
Subtle SE	13	3.7	19	4.6	0.538
*Illness severity (median, IQR)*					
STESS	3	2–4	3	2–4	0.834
Charlson Comorbidity Index	4	2–6	4	2–6	0.250
SAPS II***	45	35–56	45	34–57	0.722

IQR = interquartile range; GCS = Glasgow Coma Score (range 3–15); SE = status epilepticus; NCSE = nonconvulsive status epilepticus; STESS = Status Epilepticus Severity Score (range 0–6)[16, 17]; Charlson Comorbidity Index (range 0–37)[18]; SAPS II = Simplified Acute Physiology Score II (range 0–163)[19]

*Acute autoimmune encephalitis was defined as the presence of antigen-specific antibodies in the serum and/or cerebrospinal fluid, or cases exhibiting a clinically recognized autoimmune syndrome with supportive histopathologic evidence determined during the diagnostic workup for SE

^**^Non-fatal etiology of SE encompassed (not mutually exclusive) known epilepsy (*n* = 265), old remote ischemic or hemorrhagic strokes (*n* = 127), old remote or mild to moderate traumatic brain injury (*n* = 18), slowly growing brain tumors (*n* = 31), intoxications (*n* = 18), drug withdrawal (*n* = 75), drug side effects (*n* = 6), leukoencephalopathy (*n* = 23), brain surgery (*n* = 9), acute but small strokes (*n* = 3),

^***^available only in patients treated on the ICUs

Bold font indicates statistical significance after Bonferroni correction for multiple comparisons (set at a level of p ≤ 0.01)

### Univariable comparisons of outcomes

Table 2 presents the results of the univariable comparisons of primary (upper half) and secondary (lower half) outcomes between female and male patients.

**Table 2:** Univariable comparisons of primary and secondary and outcomes (n = 762)

Outcomes	Women (*n* = 350)		Men (*n* = 412)		
*Primary outcomes (n, %)*	*n*	%	*n*	%	*p* value
Persistent seizure termination	327	93.4	395	95.9	0.131
Return to premorbid neurologic function at discharge	150	42.9	202	49.0	0.089
In-hospital death	29	8.3	26	6.3	0.294
Death at 30 days follow-up (loss to follow-up 107 [14%])	42	12.0	36	8.7	0.085

Outcomes	Women (*n* = 350)		Men (*n* = 412)		
*Secondary outcomes*	*n*/median	%/IQR	*n*/median	%/IQR	p value
*Treatment characteristics during SE*					
Admitted via emergency medical services with SE (*n*, % of patients with out-of-hospital SE)	221	75.7	274	77.2	0.361
Seizures suspected by emergency medical services (*n*, % of cases admitted via emergency teams)	76	34.4	99	36.1	0.706
SE suspected by emergency medical services (*n*, % of cases admitted via emergency teams)	82	37.1	104	38.0	0.853
Time from alarm to hospital admission via emergency medical services (minutes; median, IQR)	52	39–67	50	37–71	0.656
Duration of in-hospital treatment (days; median, IQR)	11	6–18	10	5–18	0.124
ICU treatment (*n*, %)	185	52.9	237	57.5	0.197
Duration of ICU treatment (days; median, IQR)	3	2–8	3	2–6	0.780
Mechanical ventilation (*n*, %)	107	30.6	173	42.0	**0.001**
Duration of mechanical ventilation (days; median, IQR)	3	2–8	2	0.5–4	0.094
Number of non-anesthetic antiseizure drugs (median, IQR)	3	2–3	2	2–3	0.572
Patients with benzodiazepines as first-line antiseizure drug (*n*, %)	265	77.3	317	77.5	0.936
Patients with second-line antiseizure drugs (*n*, %)	287	83.7	327	80.0	0.189
Patients with continuous anesthetic drugs (*n*, %)	89	25.4	157	38.1	** < 0.001**
Duration of continuous anesthetics (hours; median, IQR)	30.0	12–112	18.0	7–48	0.036
Overall SE duration (days)	1	0.5–2	0.5	0.5–1	0.011
Treatment refractory SE (*n*, %)	129	36.9	150	36.4	0.898
*Treatment characteristics during RSE*					
Patients with continuous anesthetic drugs (*n*, %)	66	51.2	108	72.0	** ≤ 0.001**
Duration of continuous anesthetics (hours; median, IQR)	35.8	12–114.5	21.6	10–66.1	0.288
Mechanical ventilation (*n*, %)	64	49.6	105	97.2	**0.001**
Duration of mechanical ventilation (days; median, IQR)	3	2–10	2	2–9	0.433
*Complications during SE (n, %)*					
Infections/sepsis	50	14.3	70	17.2	0.281
Arterial hypotension requiring vasopressors	55	15.7	69	16.8	0.700
Multiorgan failure	24	6.9	37	9.0	0.282
Care withdrawal (*n*, %)					
Care withdrawal	47	13.4	45	10.9	0.290
Withdrawal due to presumed poor prognosis	38	10.9	30	7.3	0.308
Withdrawal following patient directives	8	2.3	15	3.6	0.297
*Transfer at discharge (n, %)*					
Home	104	29.7	180	43.7	** < 0.001**
Rehab	90	25.7	102	24.8	0.802
Other hospital	67	19.1	63	15.3	0.176
Nursing home or hospice	60	17.1	41	10.0	**0.004**

IQR = interquartile range; SE = status epilepticus; ICU = intensive care unit; RSE = treatment refractory status epilepticus

Bold font indicates statistical significance after Bonferroni correction for multiple comparisons (set at a level of *p* ≤ 0.01)

**Fig. 2:**
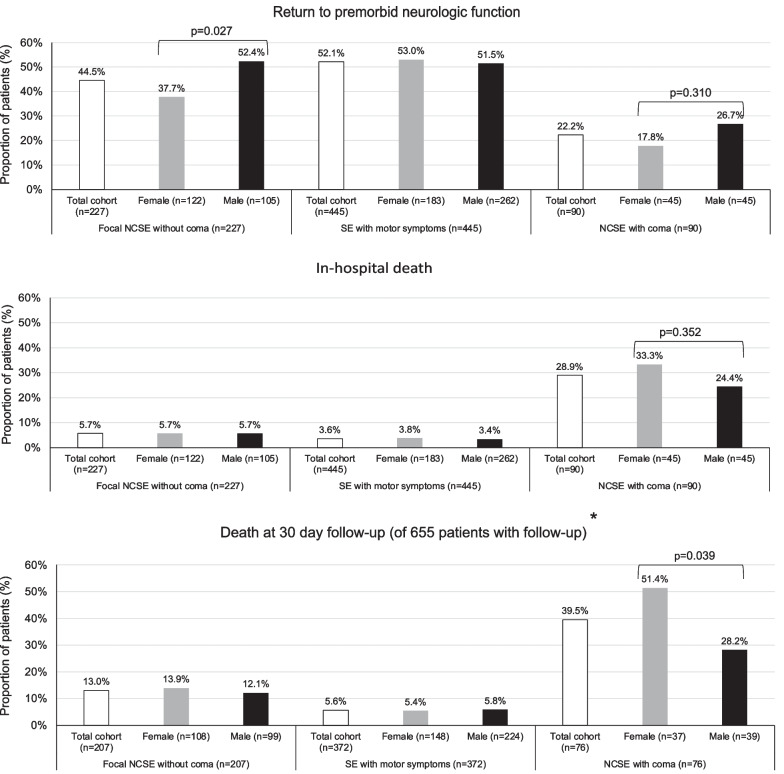
Sex-associated differences of primary outcomes among different types of status epilepticus (n = 762). SE = status epilepticus; NCSE nonconvulsive status epilepticus. *Loss to follow-up in 14% of patients

Univariable analyses revealed no significant differences of primary outcomes after Bonferroni correction for multiple comparisons (with a significant p value set at ≤ 0.01). Analyses regarding different primary outcomes of women and men in relation to specific types of SE are presented in Fig. 2. At first glance, these analyses revealed that women with focal NCSE without coma had a lower proportion of return to premorbid neurologic function as compared to men, and women with NCSE with coma had a higher proportion of death at 30 days. However, when correcting for multiple comparisons, these differences lost significance.

**Fig. 3:**
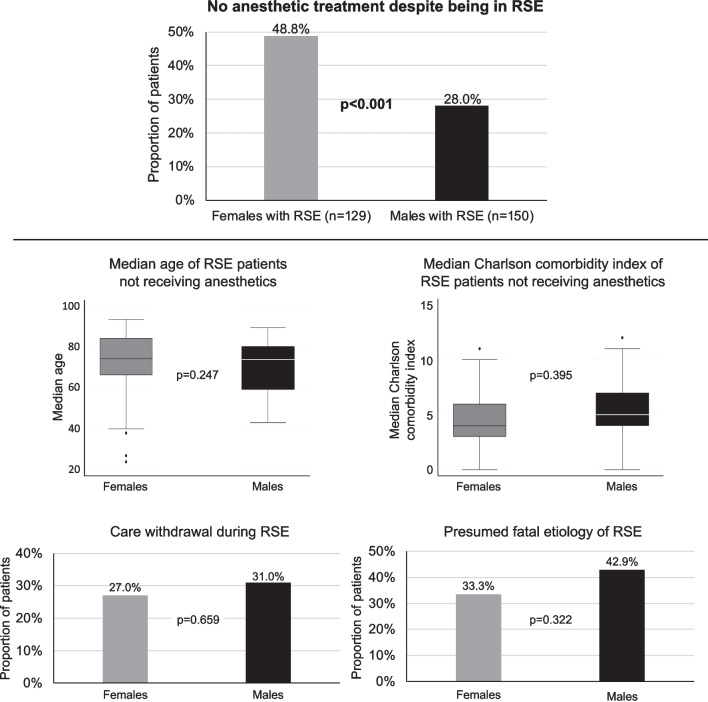
Clinical characteristics of patients with treatment refractory status epilepticus and different use of anesthetics (n = 279). RSE = treatment refractory status epilepticus; bold font indicates statistical significance after Bonferroni correction for multiple comparisons (set at a level of ≤ 0.01)

Comparisons regarding secondary outcomes revealed that women had a longer duration of SE but similar proportions of treatment refractory SE. As compared to men, women were anesthetized and mechanically ventilated less often (Table 2). Figure 3 shows subgroup analyses of patients with treatment refractory SE. These analyses revealed that women received guideline-conforming therapeutic escalation with anesthetics less often compared to men, even though there were no significant differences between men and women in terms of median age, Charlson comorbidity index, care withdrawal, and presumed fatal etiologies of the SE among patients not receiving anesthetics. Further univariable comparisons of treatment characteristics between men and women with different types of SE are presented in the Additional file 1: Table S1.

The proportion of patients with care withdrawal was comparable between men and women (Table 2). Nevertheless, women were transferred to nursing homes or hospices more frequently. Of women discharged to nursing homes or hospices 42 (70%) were > 65 years of age, 25 (41.7%) were > 80 years of age, and only 4 (6.7%) had potentially fatal etiologies and 16 (26.7%) were in treatment refractory SE that could not be terminated in 6 (10%) women.

### Uni- and multivariable logistic regression analyses regarding primary outcomes

Table 3 (upper half) shows the results of uni- and multivariable logistic regression analyses investigating the potential association between sex and outcomes while adjusting for differences between men and women as identified by the univariable comparisons and for well-established outcome predicting characteristics, such as SE severity as quantified by the STESS. These analyses found increasing age to consistently be the strongest predictor of poor outcomes, including no return to premorbid function, death during hospital stay, and death at 30 days post-SE onset independently of potential confounding factors. However, the analyses did not find an independent association between female sex and outcome. The Box-Tidwell test revealed insignificant *p* values for the interaction term of our continuous variable “age” with its corresponding natural log indicating its linear relation to all primary outcomes. The Hosmer–Lemeshow goodness-of-fit tests were all insignificant indicating adequate model fits.

**Table 3:** Univariable and multivariable logistic regression analyses regarding primary and secondary outcomes (n = 762)

Primary outcomes						
	Univariable model			Multivariable model* corrected for the influence of both centers		
Potential predictors of outcome/confounders (as identified in Table 1 and as established in the literature)	OR	95% CI	p value	OR	95% CI	p value
*Return to premorbid neurologic function*						
Female sex	0.78	0.59–1.04	0.089	0.75	0.55–1.02	0.070
Age (per every additional year of age)	0.97	0.97–0.98	** < 0.001**	0.99	0.98–0.99	**0.011**
Acute intracranial hemorrhage	0.27	0.16–0.46	** < 0.001**	0.28	0.16–0.48	** < 0.001**
SE severity (as quantified by the STESS)	0.36	0.27–0.49	** < 0.001**	0.49	0.33–0.73	** < 0.001**
SE type	0.90	0.73–1.11	0.313	1.07	0.83–1.38	0.584
*In-hospital death*						
Female sex	1.34	0.77–2.32	0.295	1.40	0.77–2.53	0.268
Age (per every additional year of age)	1.04	1.02–1.06	** < 0.001**	1.04	1.01–1.06	**0.002**
Acute intracranial hemorrhage	2.13	1.08–4.20	**0.030**	1.87	0.89–3.90	0.097
SE severity (as quantified by the STESS)	5.35	2.49–11.49	** < 0.001**	1.64	0.64–4.23	0.304
SE type	2.85	1.92–4.23	** < 0.001**	2.77	1.78–4.32	** < 0.001**
*Death at 30 days follow-up*						
Female sex	1.34	0.77–2.32	0.295	1.27	0.90–1.80	0.177
Age (per every additional year of age)	1.04	1.02–1.06	** < 0.001**	1.04	1.03–1.05	** < 0.001**
Acute intracranial hemorrhage	2.13	1.08–4.20	**0.030**	1.19	0.72–1.97	0.492
SE severity (as quantified by the STESS)	2.05	1.45–2.89	** < 0.001**	0.96	0.60–1.52	0.849
SE type	1.26	0.99–1.59	0.059	1.44	1.09–1.90	**0.010**

Secondary outcomes						
	Univariable model			Multivariable model* corrected for the influence of both centers		
Potential influences on treatment decisions (as identified in Table 1)	OR	95% CI	p value	OR	95% CI	p value
*Use of anesthetics*						
Female sex	0.55	0.41–0.76	** < 0.001**	0.64	0.45–0.91	**0.013**
Age (per every additional year of age)	0.97	0.97–0.98	** < 0.001**	0.98	0.97–0.99	** < 0.001**
Acute intracranial hemorrhage	1.50	0.96–2.34	0.073	1.32	0.78–2.21	0.299
SE type	2.77	2.18–3.52	** < 0.001**	3.35	2.57–4.37	** < 0.001**
*Use of mechanical ventilation*						
Female sex	0.61	0.45–0.82	**0.001**	0.72	0.52–1.00	**0.050**
Age (per every additional year of age)	0.98	0.97–0.99	** < 0.001**	0.98	0.97–0.99	** < 0.001**
Acute intracranial hemorrhage	2.39	1.54–3.70	** < 0.001**	2.46	1.50–4.03	** < 0.001**
SE type	2.84	2.25–3.60	** < 0.001**	3.11	2.42–3.99	** < 0.001**

OR = odds ratio; CI = confidence interval; SE = status epilepticus; STESS = status epilepticus severity score

*All Hosmer–Lemeshow goodness-of-fit tests insignificant indicating adequate model fit

Bold font indicates statistical significance (with a *p* value set at ≤0.05)

**Fig. 4:**
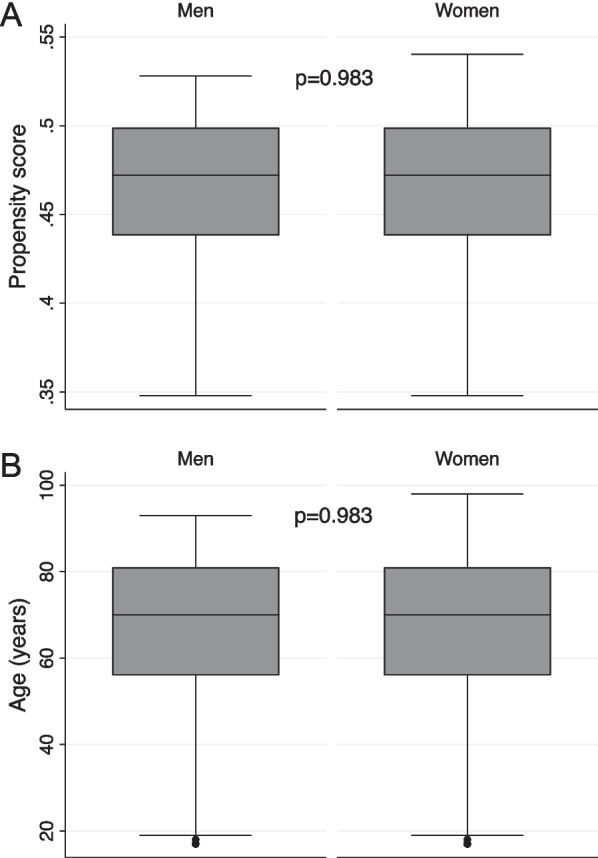
Propensity score (**A**) and age (**B**) distribution in the propensity score-matched cohort (n = 417)

Subsequently, patients were matched according to their propensity scores generated with age to account for the imbalances regarding age between men and women. Figure 4A and B presents the balanced cohort regarding the propensity score distribution and age after matching females with males according to their propensity score generated by age. While the propensity score-matched multivariable analyses revealed no association between female sex and in-hospital death or death at 30 days after seizure onset, female sex was associated with decreased odds for return to premorbid neurologic function independently of potential confounders, such as age, acute intracranial hemorrhage, STESS, and type of SE (Table 4).

**Table 4:** Univariable and multivariable logistic regression analyses regarding primary outcomes within the propensity score-matched cohort (*n* = 417)

Primary outcomes						
	Univariable model			Multivariable model* corrected for the influence of both centers		
Potential predictors of outcome/confounders (as identified in Table 1 and as established in the literature)	OR	95% CI	p value	OR	95% CI	p value
*Return to premorbid neurologic function*						
Female sex	0.62	0.46–0.84	**0.002**	0.48	0.33–0.68	** < 0.001**
Age (per every additional year of age)	0.97	0.96–0.98	** < 0.001**	0.97	0.96–0.98	** < 0.001**
Acute intracranial hemorrhage	0.07	0.03–0.16	** < 0.001**	0.07	0.03–0.15	** < 0.001**
SE severity (as quantified by the STESS)	0.44	0.33–0.61	** < 0.001**	0.99	0.62–1.56	0.949
SE type	0.89	0.72–1.09	0.262	0.90	0.70–1.16	0.406
*In-hospital death*						
Female sex	2.34	1.20–4.59	0.013	2.00	0.93–4.26	0.074
Age (per every additional year of age)	1.04	1.02–1.07	** < 0.001**	1.04	1.01–1.07	**0.005**
Acute intracranial hemorrhage	1.15	0.47–2.82	0.753	1.05	0.41–2.70	0.913
SE severity (as quantified by the STESS)	3.59	1.57–8.20	**0.002**	1.55	0.55–4.37	0.403
SE type	1.92	1.26–2.94	**0.003**	1.95	1.24–3.07	**0.004**
*Death at 30 days follow-up*						
Female sex	1.05	0.75–1.46	0.788	0.74	0.49–1.10	0.136
Age (per every additional year of age)	1.04	1.02–1.05	** < 0.001**	1.06	1.04–1.07	** < 0.001**
Acute intracranial hemorrhage	0.96	0.58–1.58	0.866	0.65	0.38–1.13	0.124
SE severity (as quantified by the STESS)	1.73	1.22–2.46	**0.002**	0.45	0.27–0.76	**0.003**
SE type	1.65	1.30–2.10	** < 0.001**	2.19	1.65–2.89	** < 0.001**

OR = odds ratio; CI = confidence interval; SE = status epilepticus; STESS = status epilepticus severity score

*All Hosmer–Lemeshow goodness-of-fit tests insignificant indicating adequate model fit

Bold font indicates statistical significance (with a *p* value set at 0.05)

### Uni- and multivariable logistic regression analyses regarding secondary outcomes

Uni- and multivariable analyses for the use of anesthetics and mechanical ventilation (defined as secondary outcomes) are presented in Table 3 (lower half). These analyses revealed that increasing age and female sex were both independently associated with decreased odds for the continuous administration of anesthetics and the use of mechanical ventilation. The Hosmer–Lemeshow goodness-of-fit tests all indicated adequate model fits.

## Discussion

This observational study investigated sex-associated differences of SE, its treatment practices, course of disease, complications, and outcomes in a large cohort of adult patients treated at two well equipped Swiss academic tertiary medical care centers. Besides the large cohort of 762 adult SE patients with similar clinical characteristics to those in other adult SE studies including age [26–30], outcome [6, 27, 31], etiologies [6, 27–29], complications [30, 31], SE severity [27, 28], and types of SE [6, 26], our study carefully accounted for the withdrawal of care due to patients’ directives or presumed poor prognosis, and for many additional potential confounders that were frequently neglected in previous studies but can significantly affect outcomes. An additional strength of the study is the utilization of propensity score-matched analyses, which account for imbalances in age distribution between men and women. While our study revealed an independent association of age with all primary outcomes, female sex was not independently associated with the primary outcomes after correcting for potential confounders in our multivariable models at first glance. However, propensity score-matched analyses accounting for potential imbalances regarding age between men and women revealed that female sex was associated with decreased odds for return to premorbid neurologic outcome independently of potential confounders. This comes along with an independent association of female sex with decreased odds for being treated with anesthesia and mechanical ventilation aside from other factors, such as age, intracranial hemorrhage, and types of SE.

The slightly lower number of women admitted to our care centers during the study period is in line with some early studies suggesting a lower incidence of SE in women than men [5–8, 32]. To what degree this lower number of women represents a true lower incidence, under-detection, or undertreatment remains unclear. While women had a higher median age, and a longer median duration of SE, most treatment characteristics did not markedly differ except for a lower percentage receiving anesthesia and mechanical ventilation. When focusing on patients who experienced treatment refractory SE, analyses revealed that women received guideline-conforming treatment escalation with anesthetics less often compared to men, even though there were no significant differences between men and women in terms of median age, Charlson comorbidity index, care withdrawal, and presumed fatal etiologies (except for intracranial hemorrhage seen more frequently in men) in the subgroup of patients with treatment refractory SE (as presented in Fig. 1). The low percentage of non-survivors who received anesthetics appears in line with previous studies from our group demonstrating how early anesthesia, in particular if directly introduced after benzodiazepines, reduced SE duration and hospital stay, improving outcome especially in the absence of potentially fatal etiologies [33]. The findings of our study regarding undertreatment of women are, unfortunately, consistent with previous studies reporting undertreatment of critically ill women in intensive care medicine [4, 34]. In a recent meta-analysis by Modra et al. including 545′538 critically ill patients admitted to ICU, women received less invasive ventilation, renal replacement therapies and had a shorter ICU stay [34]. Sociocultural differences more than biology may account for these findings. While previous studies have revealed that advanced directives and treatment limitations are more prevalent in women [35] and that female sex is a risk factor for care withdrawal or limited ICU treatments [36], we found no differences in terms of care withdrawal in our study. However, although documented care withdrawal in women did not markedly differ from withdrawal in men, women in our cohort were transferred to rehabilitation centers less often but were discharged to nursing homes or hospices more frequently. These results are all the more worrying when considering that women discharged to nursing homes or hospices in our study were > 80 years of age in only 42%, had potentially fatal etiologies in only 7%, and had refractory SE that could not be terminated in only up to 10%. The exact reasons for the higher proportion of women being transferred to nursing homes or hospices could not be clarified due to the retrospective nature of our study. However, less intensive care and/or withdrawal of care may also be a possible explanation, why in an earlier study using the Taiwan National Health Insurance Research Database, the in-hospital mortality for women with SE increased rapidly after the age of 40–45 years [32]. A rather provocative hypothesis for these findings may be that especially in older age and earlier generations, men are still less able to take care of their wives at home than vice versa, as women are still often more involved in caregiving and household chores or men where simply deceased. Effort has been made in cardiovascular research, but also during the COVID-19 pandemic, to assess the impact of sociocultural (gender) variables (e.g. marital status, responsibility in household, caregiving duties) on disease manifestations and outcomes [37, 38]. Unfortunately, studies of SE or epilepsy patients in this context are scarce and further information on sociocultural variables were not available in our study.

ICU admission is a prerequisite for treatment of patients with SE. Although women outlive men worldwide, women remain underrepresented in ICU patients [39]. Sex and gender differences in ICU admission have been reported, mostly revealing a disadvantage for women [4, 40, 41]. If the focus is shifted away from SE or epilepsy patients and toward neurocritically ill patients in general, a study of 450′948 adult patients revealed that critically ill women with cardio- and neurovascular diagnoses had a lower likelihood for ICU admission compared to men, despite being more severely ill [4]. In our study, illness severity as assessed by the SAPS II and Charlson Comorbidity Score did not differ between sexes. Not surprisingly, biological sex and sex-specific thresholds as estimates of organ dys/function (SAPS II) are not included in ICU risk assessment tools, and may thus underestimate illness severity in women, given their lower thresholds in most biomarkers. Accordingly, earlier studies have described female sex as an important promotor of the emergence of SE in patients with epilepsy [3] and that female sex is associated with a higher mortality in SE [32]. This is a serious and worrisome hypothesis which warrants further studies, including careful reassessment of contemporary risk assessment tools in order to provide equal opportunities to men and women [42].

Finally, our study revealed that women had focal NCSE without coma more, and SE with motor symptoms less often. As focal NCSE without coma represents a less severe SE type than convulsive types or NCSE with coma [16], the decreased odds for return to premorbid function in women when accounting for potential imbalances regarding age and adjusting for potential confounders are even more worrisome.

Considering our additional results, that female sex is independently associated with decreased odds for the use of anesthetics and mechanical ventilation it seems more than plausible that this undertreatment takes its toll.

The fact that women in our cohort were older than men further offers the hypothesis that young women may be hormonally protected against seizures and that protection may vanish with the age-related decrease in sexual hormones. Studies have focused on the influence of gonadal hormones on the evolution of seizures in women with epilepsy [43]. The prevailing view is that estrogen may promote the emergence of seizures whereas progesterone is thought to prohibit seizures. However, sound studies on hormonal influences regarding SE are lacking and the retrospective nature of our study did not allow for further analyses in this regard.

The only rather reassuring result in our study is the finding that, in the multivariate analysis, female sex was not an independently associated with the other primary outcomes under investigation such as in-hospital death and death at 30 days after seizure onset. The extent to which this finding downplays the aforementioned results warrants critical consideration by the reader.

The independent association of female sex and disparities regarding specific treatment and outcome after multivariable adjustments and after accounting for imbalances regarding age between men and women strongly suggest that our findings are unlikely solely explainable by age or the well-known potential confounders. However, it is essential to recognize the inherent limitations of retrospective studies when examining sex-related differences in SE management and outcomes. Although intriguing, the retrospective study design with a Swiss two center cohort does not allow firm conclusions regarding a causal relation between undertreatment and the lower odds of return to premorbid function of women.

Accordingly, we advise against drawing overly hasty conclusions or providing premature reassurances based solely on the results of this one study. Until further research provides more certainty, physicians are urged to be heighten their awareness of age-, sex- or gender-related unequal treated of such critically ill patients and to actively strive toward a more equitable and inclusive medicine, ensuring optimal SE outcomes for all individuals, irrespective of gender or age.

### Limitations

Our study has additional limitations to the ones discussed above that must be acknowledged. Firstly, our study was conducted at only two Swiss academic tertiary care centers, which limits the generalizability of our findings to other settings. Secondly, the observational study design is prone to several biases that may have been missed but have influenced our results. Thirdly, SE duration represents an approximation, particularly in cases of missing EEG data due to recording errors (in 2.1%) and/or nonconvulsive SE with unwitnessed seizure onset. In addition, potentially delayed EEG confirmation of SE termination caused by the time needed to organize and interpret the EEG may have led to an overestimation of SE duration, and the opposite is true when it comes to estimating seizure onset without motor symptoms thus requiring confirmation of suspected SE by EEG. Fourth, analyses regarding sex-related delay of treatment could not be performed as reliable data regarding the onset of SE is often unavailable. The latter is explained by the fact that the exact timepoint at which especially nonconvulsive seizures start is not precisely known (a shortcoming even hardly avoidable in prospective studies).

## Conclusion

This study has uncovered concerning sex-related disparities in the clinical characteristics, therapeutic interventions, and outcomes of adult patients experiencing SE, with women exhibiting a relative disadvantage. To what extent these results are explained by undetected and unexplored sex-specific differences in a systemic response to SE remains unclear. Nevertheless, this does not make them any less concerning. Until further studies provide more explanations in this context, these findings indicate that sex must be taken into account when formulating strategies for managing SE and forecasting specific outcomes.

### Supplementary Information

**Additional file 1**. Univariable comparison of treatment characteristics between men and women with different types of status epilepticus.

## Data Availability

The datasets used and/or analyzed during the current study are available from the corresponding author on reasonable request.

## Abbreviations

CI: Confidence interval
GCS: Glasgow coma scale
HIE: Hypoxic-ischemic encephalopathy
ICU: Intensive care unit
ILAE: International league against epilepsy
IQR: Interquartile range
NCSE: Nonconvulsive status epilepticus
OR: Odds ratio
REDCAP: Research electronic data capture
SAPS II: Simplified acute physiology scale II
SE: Status epilepticus
STESS: Status epilepticus Severity score
