# Supplementary material for: Sex-related differences in adult patients with status epilepticus: a seven-year two-center observation
Source: Crit Care. 2023 Aug 5;27:308. doi: 10.1186/s13054-023-04592-6 (PMC10403848; doi:10.1186/s13054-023-04592-6)
Supplement: Supplementary file 1 — Additional file 1. Univariable comparison of treatment characteristics between men and women with different types of status epilepticus. [file 13054_2023_4592_MOESM1_ESM.docx]

**Supplemental table 1:** Univariable comparison of treatment characteristics between men and women with different types of status epilepticus

| **Focal NCSE without coma** | **Women**  **(n=120)** | | **Men**  **(n=102)** | |  |
| --- | --- | --- | --- | --- | --- |
| **🡪 After excluding patients with care withdrawal following**  **patients’ directives** | **n / median** | **% / IQR** | **n / median** | **% / IQR** | **p-value** |
| Duration of in-hospital treatment (days; median, IQR) | 13 | 8-22 | 11 | 6-20 | 0.085 |
| ICU treatment (n, %) | 45 | 37.5 | 34 | 33.3 | 0.518 |
| Duration of ICU treatment (days; median, IQR) | 3 | 2-9 | 3 | 2-7 | 0.934 |
| Mechanical ventilation (n, %) | 13 | 10.8 | 11 | 10.8 | 0.991 |
| Duration of mechanical ventilation (days; median, IQR) | 0.5 | 0-1 | 0.5 | 0-0.5 | 0.841 |
| Number of non-anesthetic antiseizure drugs (median, IQR) | 3 | 2-4 | 2 | 2-3 | **0.028** |
| Benzodiazepines as first-line antiseizure drug (n, %) | 85 | 70.8 | 73 | 71.6 | 0.813 |
| Second-line antiseizure drugs (n, %) | 108 | 90.0 | 91 | 89.2 | 0.980 |
| Continuous anesthetic drugs (n, %) | 14 | 11.7 | 10 | 9.8 | 0.656 |
| Duration of continuous anesthetics (hours; median, IQR) | 74.3 | 21.3-128 | 43.4 | 16.7-159 | 0.738 |
| **SE with motor symptoms** | **Women**  **(n=178)** | | **Men**  **(n=252)** | |  |
| **🡪 After excluding patients with care withdrawal following**  **patients’ directives** | **n / median** | **% / IQR** | **n / median** | **% / IQR** | **p-value** |
| Duration of in-hospital treatment (days; median, IQR) | 10 | 4-15 | 8 | 5-14 | 0.625 |
| ICU treatment (n, %) | 101 | 56.7 | 158 | 62.7 | 0.214 |
| Duration of ICU treatment (days; median, IQR) | 3 | 2-5 | 3 | 2-4.5 | 0.609 |
| Mechanical ventilation (n, %) | 68 | 38.2 | 131 | 52.0 | **0.005** |
| Duration of mechanical ventilation (days; median, IQR) | 0.5 | 0-2 | 0.5 | 0-2 | 0.300 |
| Number of non-anesthetic antiseizure drugs (median, IQR) | 2 | 2-3 | 2 | 2-3 | 0.113 |
| Benzodiazepines as first line antiseizure drug (n, %) | 143 | 80.3 | 205 | 81.3 | 0.991 |
| Second-line antiseizure drugs (n, %) | 134 | 80.3 | 184 | 73.0 | 0.446 |
| Continuous anesthetic drugs (n, %) | 56 | 31.5 | 116 | 46.0 | **0.002** |
| Duration of continuous anesthetics (hours; median, IQR) | 19.9 | 7.3-39 | 14.1 | 6.9-32.5 | 0.112 |
| **NCSE with coma** | **Women**  **(n=44)** | | **Men**  **(n=43)** | |  |
| **🡪 After excluding patients with care withdrawal following**  **patients’ directives** | **n / median** | **% / IQR** | **n / median** | **% / IQR** | **p-value** |
| Duration of in-hospital treatment (days; median, IQR) | 17 | 7-27 | 18 | 10-31 | 0.195 |
| ICU treatment (n, %) | 36 | 81.2 | 39 | 90.7 | 0.230 |
| Duration of ICU treatment (days; median, IQR) | 8.5 | 5-15 | 7 | 4-17 | 0.906 |
| Mechanical ventilation (n, %) | 24 | 54.6 | 28 | 65.1 | 0.315 |
| Duration of mechanical ventilation (days; median, IQR) | 3 | 0.5-12 | 3 | 0.5-14 | 0.376 |
| Number of non-anesthetic antiseizure drugs (median, IQR) | 3 | 2-4 | 4 | 2-4 | **0.018** |
| Benzodiazepines as first line antiseizure drug (n, %) | 32 | 72.7 | 26 | 74.4 | 0.109 |
| Second-line antiseizure drugs (n, %) | 39 | 88.6 | 39 | 90.7 | 0.665 |
| Continuous anesthetic drugs (n, %) | 17 | 38.6 | 28 | 65.1 | **0.013** |
| Duration of continuous anesthetics (hours; median, IQR) | 118.8 | 47.7-129 | 55.2 | 31.6-201 | 0.830 |

IQR = interquartile range; SE = status epilepticus ; NCSE = nonconvulsive status epilepticus; ICU = intensive care unit

**Bold font** indicates statistical significance
